# Synthesis and Biological Evaluation of Amidinourea Derivatives against Herpes Simplex Viruses

**DOI:** 10.3390/molecules26164927

**Published:** 2021-08-14

**Authors:** Anita Toscani, Rossana Denaro, Sergio Fernando Castillo Pacheco, Matteo Biolatti, Silvia Anselmi, Valentina Dell’Oste, Daniele Castagnolo

**Affiliations:** 1 School of Cancer and Pharmaceutical Sciences, King’s College London, London WC2R 2LS, UK; anita.1.toscani@kcl.ac.uk (A.T.); roxdenaro@gmail.com (R.D.); silvia.anselmi@kcl.ac.uk (S.A.); 2 Department of Public Health and Pediatric Sciences, University of Turin, 10124 Turin, Italy; sergiofernando.castillopacheco@unito.it (S.F.C.P.); matteo.biolatti@unito.it (M.B.)

**Keywords:** amidinourea, herpes simplex virus, guanidine, antivirals, amidines

## Abstract

Current therapy against herpes simplex viruses (HSV) relies on the use of a few nucleoside antivirals such as acyclovir, famciclovir and valacyclovir. However, the current drugs are ineffective against latent and drug-resistant HSV infections. A series of amidinourea compounds, designed as analogues of the antiviral drug moroxydine, has been synthesized and evaluated as potential non-nucleoside anti-HSV agents. Three compounds showed micromolar activity against HSV-1 and low cytotoxicity, turning to be promising candidates for future optimization. Preliminary mode of action studies revealed that the new compounds act in an early stage of the HSV replication cycle, just after the viral attachment and the entry phase of the infection.

## 1. Introduction

**Figure 1:**
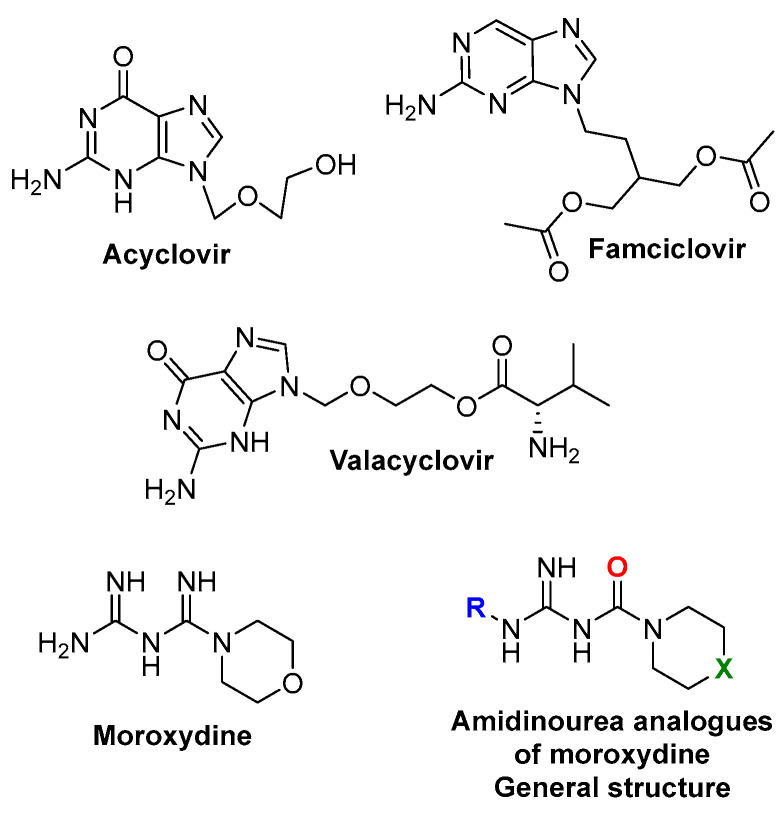
Antivirals active against HSV and the general structure of the amidinourea antivirals discussed in this work.

Herpes simplex viruses type 1 (HSV-1) and type 2 (HSV-2) are double-stranded (ds) DNA, nuclear-replicating and enveloped viruses, members of the *Alphaherpesvirinae* subfamily. They are associated with a number of clinical manifestations in humans, including cold sores, keratitis, meningitis and encephalitis as well as various other diseases in immunocompromised patients [1,2,3]. They are considered to be the most common pathogens affecting humans, as it has been calculated that 50–90% of the worldwide population is seropositive to HSV-1 [4,5]. One of the main problems with HSV is the ability of the viruses to become latent in sensory neurons and reactivate periodically, causing lesions at the original site of the primary infection, for instance, in the mouth or on the genitals [6]. It has been estimated than nearly 490 million people between 15 and 49 years of age live with an infection of HSV-2, which is the main cause of genital herpes [7]. HSV prevalence has been increasing in the last 20 years worldwide [8,9] and HSV latent infections can cause lifelong major health problems to the host. The viral DNA can stay latent in the nucleus of sensory neurons and avoid the host’s immune system, leading, in the long term, to life-threatening diseases such as neurological disorders, encephalitis and blindness [8,9]. Recently, a link between the infections of HSV-1 and neurodegenerative diseases such as Alzheimer’s disease has been demonstrated [10,11,12]. The standard therapy against HSV infections relies on the use of the antiviral acyclovir and related nucleoside analogues, such as famciclovir and valacyclovir (Figure 1), while no effective vaccine is currently available [13,14]. Although acyclovir is effective against primary infections, it proved to be ineffective against the latent virus and thus unable to completely eradicate the infection from the host. In addition, the nephrotoxicity associated with acyclovir and the emergence of drug-resistant HSV strains [15,16,17] highlights the need to identify new anti-HSV drugs, possibly working with a different mode of action. Amidinoureas are a class of organic compounds that find application in medicinal chemistry as antifungal, antibacterial and anticancer agents [18,19,20,21,22,23]. Recently, our group reported the synthesis of a series of amidinourea derivatives, designed as analogues of the antiviral drug moroxydine, an inhibitor of hepatitis C virus (HCV) [24,25]. These molecules seem to act against HCV virus through a mode of action that differs from that of nucleoside-based antivirals, namely the inhibition of virus translation.

Driven by our interest on amidinoureas and antimicrobial agents, we decided to further investigate the antiviral properties of amidinourea analogues of moroxydine against DNA viruses. Herein, we describe the synthesis of a series of amidinourea derivatives, designed as isosters of moroxydine, having the general structure reported in Figure 1 and their evaluation against HSV-1 and HSV-2.

## 2. Results and Discussion

**Scheme 1:**
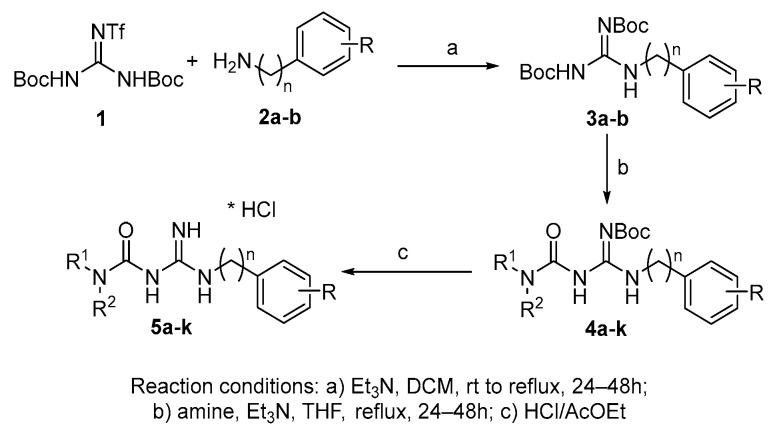
Synthesis of amidinourea derivatives **5a**–**k**.

A library of amindinourea compounds was synthesized as shown in Scheme 1 [24].

**Figure 2:**
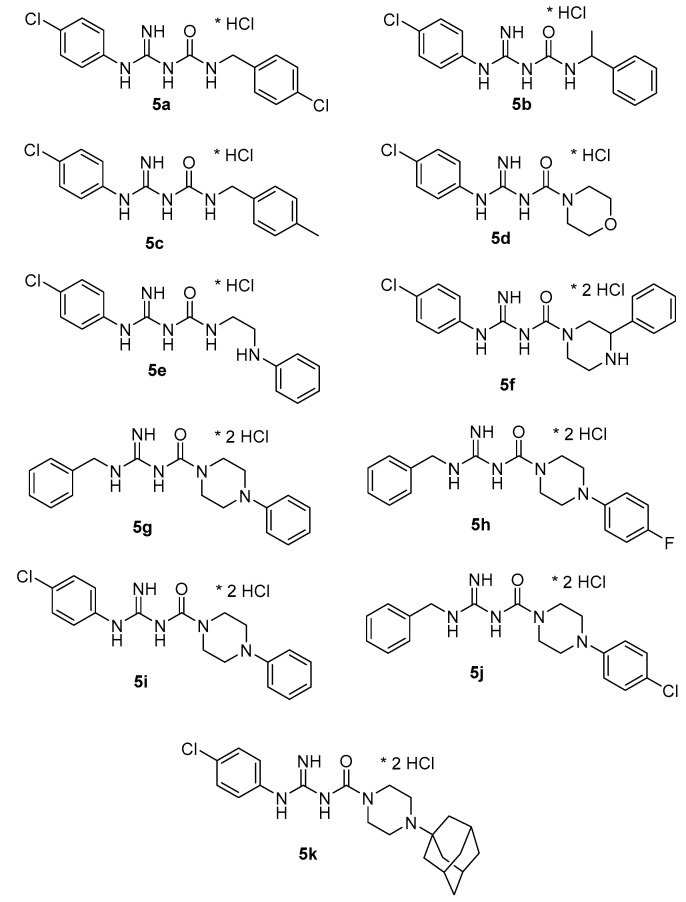
Structures of the amidinourea derivatives **5a**–**k**.

Benzyl amine and p-Cl-aniline were reacted with 1,3-di-Boc-2-(trifluoromethylsulfonyl) guanidine **1** in DCM and in the presence of Et_3_N affording the substituted guanidines **3a**–**b** in high yields. These latter were then reacted with various primary and secondary amines in refluxing THF to afford the desired Boc-protected amidinoureas **4a**–**k** after 24–48 h. The deprotection of **4a**–**k** was carried out in HCl/AcOEt leading to desired amidinoureas **5a**–**k** as hydrochloride salts in high yields (Figure 2). Full details regarding preparation procedures and characterization data are included in the Supplementary Materials.

We then investigated whether the new amidinourea derivatives **5a**–**k** could affect HSV-1 and HSV-2 replication. We first assessed cell cytotoxicity of the compounds in African green monkey kidney cells (Vero) to rule out the possibility that antiviral activity was related to the cytotoxic effects of the drug (Table 1). Vero cells were seeded at a density of 3 × 10^4^/well in a 96-well culture plate. After 24 h, the cells were treated with different dilutions of drugs, or mock-treated using the same amount of vehicle solution (DMSO). Forty-eight hours after treatment, cell viability was determined using a standard 3-(4,5-dimethylthiazol-2-yl)-2,5-diphenyltetrazolium bromide (MTT) assay. The 50% cytotoxic concentration (CC_50_) values are reported in Table 1. The compounds were considered non-toxic only if they maintained at least 70% cell viability after 48 h of treatment.

**Figure 3:**
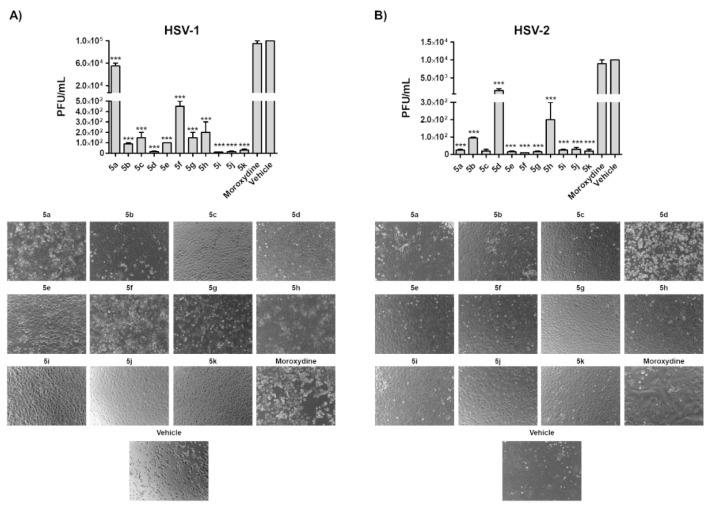
Antiviral activity of **5a**–**k** against HSV-1 and HSV-2. Upper panel. Virus yield reduction assay. Vero cells were treated as described in the manuscript with the indicated compounds. The extent of (**A**) HSV-1 and (**B**) HSV-2 replication was then assessed by titrating the infectivity of supernatants and cell-associated viruses using a standard plaque assay in Vero cells. Plaques were microscopically counted, and the mean plaque counts for each molecule were expressed as PFU/mL. Mean ± SEM is shown (***, *p* < 0.001, one-way ANOVA followed by Bonferroni’s post-tests). Lower panel. Representative light microscopy images of Vero cells infected with (**A**) HSV-1 or (**B**) HSV-2 and treated with the indicated compounds, as described for virus yield reduction assay. Original magnification: 20×.

Amidinourea **5a**–**k** derivatives were then evaluated for their antiviral activity against HSV-1 and HSV-2 at the minimum effective dose that did not show particular cytotoxicity (100 µM for **5a**–**e** and **5g**–**j**; 50 µM for **5f** and **5k**, based on MTT results). Data are reported in Figure 3. Vero cells were seeded at a density of 3 × 10^5^/well in a 24-well culture plate. Twenty-four hours later, the cells were pre-treated with the different drugs for 2 h at 37 °C, and then infected in duplicate with HSV-1 or HSV-2 at an MOI of 0.1, in the presence of the compounds; following virus adsorption, the viral inoculum was removed and cultures were exposed to the compounds for 48 h. The extent of HSV-1 and HSV-2 replication was then assessed by titrating the infectivity of supernatants and cell-associated viruses using a standard plaque assay in Vero cells. Plaques were microscopically counted, and the mean plaque counts for each drug concentration were expressed as plaque-forming units per milliliter (PFU/mL) (Figure 3). All the compounds inhibited both HSV-1 and HSV-2 replication by over 90%. Interestingly, the compounds **5i**, **5j** and **5k** showed a very strong activity against both HSV-1 and HSV-2. The antiviral activity of the compound **5d**, isostere of moroxydine, is more pronounced against HSV-1, while compounds **5a**–**c** and **5e**–**f** are more active against HSV-2. It is noteworthy that moroxydine does not show any antiviral activity against both HSV-1 and HSV-2 strains (Figure 3).

**Figure 4:**
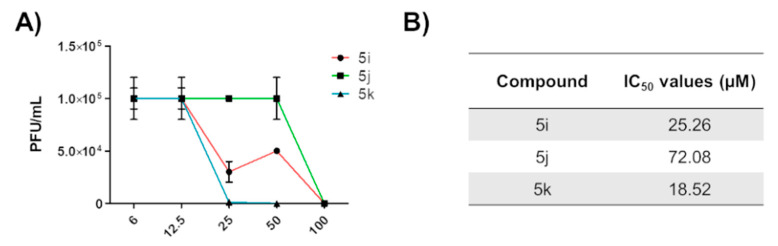
(**A**) Vero cells were infected with HSV-1 and, where indicated, the cells were treated with increasing concentrations of the indicated compounds, before and during virus adsorption. These remained in the culture media throughout the experiment. The extent of HSV-1 replication was then assessed by titrating the infectivity of supernatants and cell-associated viruses—obtained from freeze (liquid nitrogen)/thaw (37 °C) cycles—and combined using a standard plaque assay. Plaques were microscopically counted, and the mean plaque counts for each drug concentration were expressed as PFU/mL. The number of plaques was plotted as a function of drug concentration. (**B**) IC_50_ values, i.e., compound concentration required to inhibit 50% of viral infectivity.

For the more promising compounds **5i**, **5j** and **5k**, a more detailed analysis with serial dilutions of the compounds revealed that they specifically inhibited HSV-1 replication in a dose-dependent manner (Figure 4A), with the half maximal inhibitory concentration (IC_50_) values of 25.26, 72.08 and 18.52 μM, respectively (Figure 4B).

**Figure 5:**
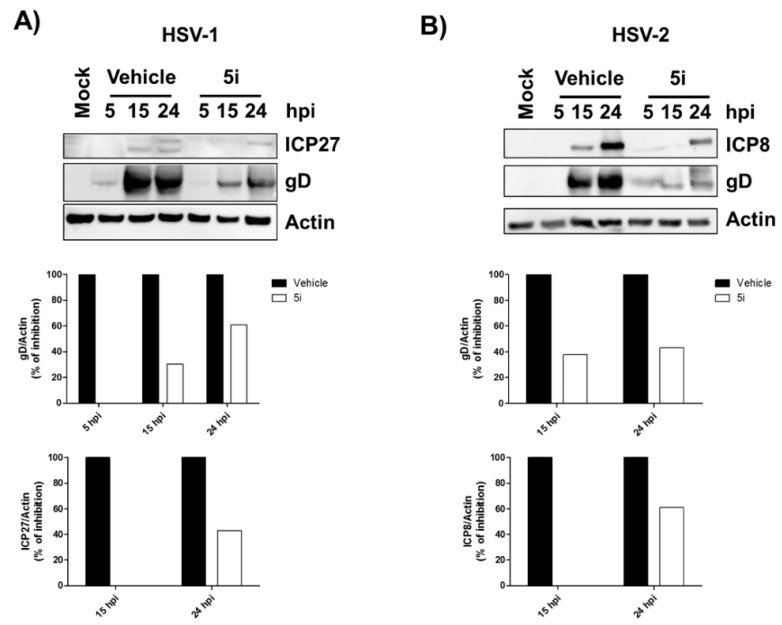
Vero cells were mock infected or infected with HSV-1 (**A**) or HSV-2 (**B**) at an MOI of 0.3. Protein levels of ICP27 (HSV-1), ICP8 (HSV-2) and gD (HSV-1/2) were assessed at different times post infection (hpi) by Western blotting (upper panels). Viral proteins were subjected to densitometry and normalized to Actin, serving as a loading control (lower panels).

Finally, to identify the phase of HSV replication program that is affected by amidinourea derivatives, the effects of **5i** compound on viral gene expression was investigated in HSV-1 and HSV-2-infected cells. To this end, the total protein cell extracts were prepared from HSV-infected Vero cells treated with **5i**, or vehicle control, for various lengths of time post infection. The expression patterns of ICP27, for HSV-1, ICP8, for HSV-2, gD, for HSV-1 and HSV-2, were then examined via immunoblotting with specific antibodies and were used as a reflection of the levels of immediate-early/early (ICP27, ICP8) and late (gD) viral products, respectively. As shown in Figure 5, an overall decrease of viral proteins was observed at every time point. These results indicate that **5i** interferes with a molecular event that occurs in an early stage of the HSV replication cycle, following the viral attachment and entry phases. However, the precise mechanism exploited by amidinourea derivatives to carry out their antiviral activity against HSV still deserves to be elucidated and is beyond the goals of the present communication.

## 3. Materials and Methods

### 3.1. Chemistry-General Methods

NMR spectra were recorded on Bruker Ascend™ 400 MHz spectrometer. ^1^H and ^13^C spectra were referenced relative to the solvent residual peaks and chemical shifts (δ) reported in ppm downfield of trimethylsilane (CDCl_3_ δ H: 7.26 ppm, δ C: 77.0 ppm; CD_3_OD-*d*_4_ δ H: 3.31 ppm, δ C: 49.05 ppm; DMSO-*d*_6_ δ H: 2.50 ppm, δ C: 39.52 ppm). Coupling constants (J) are reported in Hertz and rounded to 0.5 Hz. Splitting patterns are abbreviated as follows: singlet (s), doublet (d), triplet (t), quartet (q), multiplet (m), broad (br) or some combination of these. Thin-layer chromatography (TLC) was performed using commercially available precoated plates and visualized with UV light at 254 nm; KMnO_4_ or ninhydrin dips were used to reveal the products. Flash column chromatography were carried out using Aldrich Chemistry 40–63 µm 60 Å. All solvents and commercially available reagents were used as received. All chemicals and solvents were used as supplied, unless noted otherwise. The amount of compounds obtained as oils was quantified by weight. The isolated clean fraction of the oils resuspended in dichloromethane was dried using rotary evaporation first, and then a high-vacuum pump. Full characterization of compounds **4**–**5** is reported in the Supporting Information.

### 3.2. Biology

#### 3.2.1. Cells and Viruses

The African green monkey kidney cells (Vero, ATCC CCL-81™) were cultured in Dulbecco’s Modified Eagle’s Medium, supplemented with 10% heat inactivated fetal bovine serum (FBS), 2 mM glutamine, 1 mM sodium pyruvate, 100 U/mL penicillin and 100 µg/mL streptomycin sulfate (Sigma-Aldrich, Milan, Italy). Clinical isolates of HSV-1 and HSV-2 were a generous gift from Valeria Ghisetti, Amedeo di Savoia Hospital, Turin, Italy. They were propagated and titrated by plaque assay on Vero cells, as previously described [26].

#### 3.2.2. Virus Yield Reduction Assay

Vero cells were seeded in 24-well plates and pre-treated, after 24 h, with different concentrations of the amidinourea derivatives or the vehicle control (DMSO) for 2 h at 37 °C. They were then infected with HSV at MOI of 0.1 PFU/cell in the presence of the compounds. Following virus adsorption (2 h at 37 °C), the viral inoculum was removed, and the cultures were maintained in a medium that contained the corresponding molecule for 48 h. The cells and supernatants were then harvested and disrupted using three freeze (liquid nitrogen)/thaw (37 °C) cycles. The extent of virus replication was subsequently assessed by titrating the infectivity of the supernatants of the cell suspensions in Vero cells, as previously described [26]. Plaques were microscopically counted, and the mean plaque counts for each drug concentration were expressed as PFU/mL.

#### 3.2.3. Cytotoxicity Assay

To determine the cytotoxicity of amidinourea derivatives, Vero cells were seeded in a 96-well culture plate and exposed to increasing concentrations of either compounds or vehicle (DMSO) the following day. After 48 h of incubation, the number of viable cells was determined using the 3-(4,5-dimethylthiazol-2-yl)-2,5-diphenyltetrazolium bromide (MTT) (Sigma-Aldrich, Milan, Italy) assay, as previously described [27].

#### 3.2.4. Western Blot Analysis

After treatment, the cells were washed with phosphate-buffered saline (PBS), and cell lysis was carried out using radioimmunoprecipitation assay (RIPA) buffer to obtain total cell lysate. An equal amount of the cell extracts was fractionated by electrophoresis on sodium dodecyl sulfate polyacrylamide gels and transferred to Immobilon-P membranes (Bio-Rad, Milan, Italy). After blocking with 5% nonfat dry milk in TBS-Tween 0.05%, the membranes were incubated overnight at 4 °C with the appropriate primary antibodies. The following primary antibodies were used: mouse monoclonal antibodies anti-HSV-1 ICP27 monoclonal antibody (MAb) (clone H1113, Virusys Corporation), mouse anti-HSV-2 ICP8 MAb (clone 4E6, Virusys Corporation), mouse anti-HSV-1/2 gD MAb (clone 2C10, Virusys Corporation) and anti-Actin (clone C4, MAB1501) (Sigma-Aldrich, Milan, Italy) (all at 1:1000 dilution in 5% nonfat dry milk, TBS-Tween 0.05%). After washing with TBST buffer (500 mM NaCl, 20 mM Tris pH 7.4, 0.05% Tween 20), the membrane was incubated with an HRP-conjugated anti-mouse secondary antibody (GE Healthcare, Chicago, IL, USA) for 1 h at room temperature, and visualized using an enhanced chemiluminescence detection kit (SuperSignal West Pico Chemiluminescent Substrate, Thermo SCIENTIFIC, Waltham, MA, USA). Scanning densitometry of the bands was performed using Images Lab software (Bio-Rad, Hercules, CA, USA).

#### 3.2.5. Statistical Analysis

Data were presented as the mean value and standard error of the mean (SEM). Data were analyzed by means of GraphPad Prism 5 software (GraphPad Software, Inc., La Jolla, CA, USA). Statistical analysis was performed using one-way ANOVA with Bonferroni’s post-test; *p* < 0.05 was considered statistically significant. The IC_50_ e CC_50_ values were calculated using the online tool Quest Graph™ IC_50_ Calculator, AAT Bioquest, Inc., Sunnyvale, CA, USA, https://www.aatbio.com/tools/ic50-calculator (accessed on 4 May 2021).

## 4. Conclusions

In conclusion, a series of amidinourea compounds has be designed using the moroxydine scaffold as a template. The new compounds have been evaluated for their activity against HSV-1 and HSV-2 showing a good antiviral activity at micromolar concentrations in the case of derivatives **5i**, **5j** and **5k**. These three most active amidinoureas also showed low cytotoxicity in Vero cells, resulting in promising hit compounds for following structure optimization studies. Early studies to elucidate the mode of action have been carried out. Being non-nucleoside inhibitors of HSV-1, it is likely that the new amidinourea compounds may act with an antiviral mechanism different from that of classic anti-HSV drugs such as acyclovir. Preliminary studies indicate that 5i inhibits HSV at the early stage of replication, in line with previous observations on the antiviral activity of amidinourea compounds on other viruses [25].

## Figures and Tables

**Table 1:** Cytotoxicity of the amidinourea derivatives **5a**–**k**.

Compound	CC_50_^1^ Values (µM)
**5a**	168.498
**5b**	196.402
**5c**	184.303
**5d**	1557.984
**5e**	142.274
**5f**	98.575
**5g**	245.538
**5h**	388.580
**5i**	149.218
**5j**	176.528
**5k**	86.733

^1^ CC_50_, compound concentration that produces 50% of cytotoxicity, as determined by MTT assays in Vero cells.

## Data Availability

Not applicable.

## Supplementary Materials

The following are available online: full characterization of compounds **4**–**5** (^1^H-NMR and ^13^C-NMR, MS).

## Sample Availability

Samples of the compounds are available from the authors.
