# Supplementary material for: Synthesis and Biological Evaluation of Amidinourea Derivatives against Herpes Simplex Viruses
Source: Molecules. 2021 Aug 14;26(16):4927. doi: 10.3390/molecules26164927 (PMC8398393; doi:10.3390/molecules26164927)
Supplement: Supplementary file 1 [file molecules-26-04927-s001.zip › molecules-1318338-supplementary.pdf]

Synthesis and biological evaluation of amidinourea derivatives against Herpes Simplex Viruses.

Anita Toscani,[1] Rossana Denaro,[1] Sergio Fernando Castillo Pacheco,[2] Matteo Biolatti,[2] Silvia Anselmi,[1] Valentina Dell'Oste, [2,*] Daniele Castagnolo[1,*]

[1]*School of Cancer and Pharmaceutical Sciences, King's College London, 150 Stamford Street, SE1 9NH, London, United Kingdom;* [2]*Department of Public Health and Pediatric Sciences, University of Turin, Turin, Italy.*

SUPPORTING INFORMATION

General Procedure for the synthesis of guanidines 3a-b

Guanidines **3a-b** have been synthesised as previously described in literature.[1]

Into a round bottom flask, the 1,3-di-Boc-2-trifluoromethylsulfonyl guanidine (1 mmol) was dissolved in DCM (5 mL). The appropriate aniline (1 mmol) and Et_3N (2 mmol) were added and the resulting mixture was stirred at room temperature for 1 h and then refluxed for 24-48 h. The reaction was monitored by TLC and stopped upon consumption of the starting material. mixture was diluted with DCM and washed once with HCl 0.1 N and once with brine. The organic extract was dried over $MgSO_4$, filtered and concentrated under reduced pressure to yield guanidines **3a-b** which proved to be pure enough to be used in the next steps without any further purification. ^{1}H-NMR and ^{13}C-NMR data are identical with those described in the literature.[1]

General procedure for the synthesis of Boc-amidinoureas 4a-k

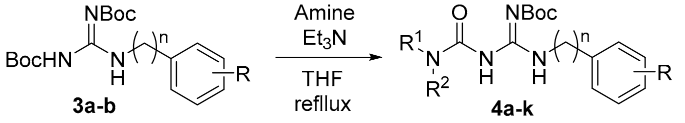

The appropriate guanidine **3a-b** (0.5 mmol) was dissolved in 5 mL of THF. The appropriate amine (1 mmol) and Et_3N (2 mmol) were added to the solution and the resulting mixture was refluxed for 24-48h. The solvent was then removed under reduced pressure and the residue was purified by flash chromatography (Hexane/AcOEt 9:1) affording the desired amidinoureas **4a-k**.

Boc-amidinourea 4a

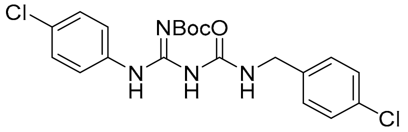

Yield: 51% (111 mg). Pale oil. **^{1}H NMR** (400 MHz, $CDCl_3$): δ 12.21 (br. s., 1H, N*H*), 10.17 (br. s., 1H, N*H*), 7.46 - 7.51 (m, 2H, *Phenyl*), 7.20 - 7.29 (m, 6H, *Phenyl*), 5.64 (br. s., 1H, N*H*), 4.35 (s, 2H, CH_2), 1.49 (s, 9H, C(CH_3)$_3$) ppm. **^{13}C NMR** (101 MHz, $CDCl_3$): δ 153.4 (C=O), 151.9 (C=N), 137.4, 133.0, 128.8, 128.7, 128.6, 128.5, 128.1, 123.5, 123.4, 83.4 (*C*(CH_3)$_3$), 43.4 (*C*H_2), 28.0 (C(*C*H_3)$_3$) ppm. **LRMS** *m/z* (ES+) m/z: 459 $[M+Na]^+$.

Boc-amidinourea 4b

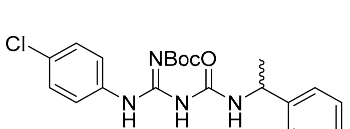

Yield: 38% (79 mg). oil. **^{1}H NMR** (400 MHz, $CDCl_3$): δ 12.19 (br. s., 1H, N*H*), 10.09 (br. s., 1H, N*H*), 7.42 -7.46 (m, 2H, *Phenyl*), 7.27 (s, 3H, *Phenyl*), 7.16 - 7.22 (m, 4H, *Phenyl*), 4.87 (m, 1H, C*H*), 1.40 - 1.43 (m, 12H, CH_3 + C(CH_3)$_3$) ppm. **^{13}C NMR** (101 MHz, $CDCl_3$): δ 163.4 (C=O), 153.4 (C=O), 151.8 (C=N), 143.9, 135.8, 129.4, 128.7, 128.6, 127.1, 125.9, 125.5, 123.3, 83.2 (*C*(CH_3)$_3$), 49.7 (*C*H), 28.0 (C(*C*H_3)$_3$), 22.5 (*C*H_3) ppm. **LRMS** *m/z* (ES+) m/z: 439 $[M+Na]^+$.

Boc-amidinourea 4c

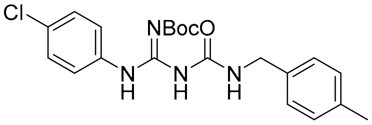

Yield: 44% (91.5 mg). Pale oil. **^{1}H NMR** (400 MHz, $CDCl_3$): δ 12.30 (br. s., 1H, N*H*), 10.17 (br. s., 1H, N*H*), 7.51 (d, *J* = 8.80 Hz, 2 H, *Cl-Phenyl*), 7.24 (s, 2H, *Phenyl*), 7.18 - 7.22 (m, 2H, *Phenyl*), 7.12 - 7.16 (m, 2H, *Phenyl*), 5.59 (br. s., 1H), 4.37 (s, 2H, CH_2), 2.33 (s, 3H, CH_3), 1.51 (s, 9H, $C(CH_3)_3$) ppm. **^{13}C NMR** (101 MHz, $CDCl_3$): δ 153.4 (C=O), 151.7 (C=N), 137.0, 135.6, 129.3, 128.7, 127.6, 126.8, 123.2, 83.3 ($C(CH_3)_3$), 43.9 (CH_2), 28.0 ($C(CH_3)_3$), 21.1 (CH_3) ppm. **LRMS** *m/z* (ES+) m/z: 439 $[M+Na]^+$.

Boc-amidinourea 4d

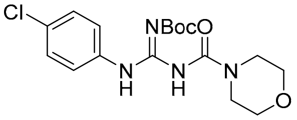

Yield: 44% (84 mg). Pale oil. **^{1}H NMR** (400 MHz, $CDCl_3$): δ 12.33 (br. s., 1H, N*H*), 10.14 (br. s., 1H, N*H*), 7.42 (d, *J* = 8.80 Hz, 2H, *Cl-Phenyl*), 7.22 - 7.27 (d, *J* = 8.80 Hz, 2H, *Cl-Phenyl*), 3.50 - 3.68 (m, 8H, *4x*CH_2 morpholine), 1.45 - 1.49 (m, 9H, $C(CH_3)_3$) ppm. **^{13}C NMR** (101 MHz, $CDCl_3$): δ 162.7 (C=O), 153.5 (C=O), 152.0 (C=N), 135.8, 129.6, 128.7, 123.5, 83.3 ($C(CH_3)_3$), 66.9 (CH_2 morpholine), 66.8 (CH_2 morpholine), 45.1 (CH_2 morpholine), 42.7 (CH_2 morpholine), 28.0 ($C(CH_3)_3$) ppm. **LRMS** *m/z* (ES+) m/z: 405 $[M+Na]^+$.

Boc-amidinourea 4e

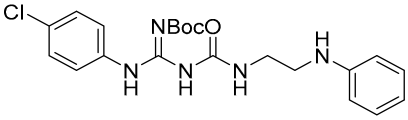

Yield: 51% (110 mg). Dark oil. **^{1}H NMR** (400 MHz, $CDCl_3$): δ 12.15 (br. s., 1H, N*H*), 10.17 (br. s., 1H, N*H*), 7.49 (d, *J* = 7.98 Hz, 2H, *Cl-Phenyl*), 7.28 (s, 2H, *Phenyl*), 7.14 - 7.22 (m, 2H, *Phenyl*), 6.72 (t, *J*=7.34 Hz, 1H, *Phenyl*), 6.58 - 6.66 (m, 2H, *Phenyl*), 5.59 (br. s., 1H, N*H*), 3.46 (m, 2H, CH_2NHC=O), 3.27 - 3.33 (m, 2H, CH_2NHPh), 1.53 (s, 9H, $C(CH_3)_3$) ppm. **^{13}C NMR** (101 MHz, $CDCl_3$): δ 153.4 (C=O), 151.8 (C=O), 147.8 (C=N), 135.6, 129.7, 129.3, 128.8, 123.7, 123.5, 117.7, 112.8, 83.5 ($C(CH_3)_3$), 44.1 (CH_2NHC=O), 39.6 (CH_2NHPh), 28.0 ($C(CH_3)_3$) ppm. **LRMS** *m/z* (ES+) m/z: 454 $[M+Na]^+$.

Boc-amidinourea 4f

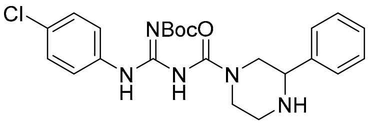

Yield: 65% (148.5 mg). Pale oil. **^{1}H NMR** (400 MHz, $CDCl_3$): δ 12.47 (d, *J* = 18.43 Hz, 1H, N*H*), 10.16 (d, *J*=18.43 Hz, 1H, N*H*), 7.41 - 7.52 (m, 3H, *Phenyl*), 7.27 - 7.40 (m, 5H, *Phenyl*), 7.19 (m, 1H, *Phenyl*), 4.62 (br. s., 1H, N*H*), 4.35 - 4.52 (m, 1H, C*H*-Ph), 3.71 (dt, *J* = 10.43, 3.54 Hz, 1H, C*H*-piperazine), 3.14 (d, *J* = 9.63 Hz, 1H, C*H*-piperazine), 2.99 - 3.10 (m, 1H, C*H*-piperazine), 2.83 - 2.96 (m, 2H, CH_2-piperazine), 2.68 - 2.83 (m, 1H, C*H*-piperazine), 1.51 (d, *J* = 1.74 Hz, 9H, $C(CH_3)_3$) ppm.

^{13}**C NMR** (101 MHz, $CDCl_3$): δ 162.5 (C=O), 162.4 (C=O), 153.5 (C=N), 151.9, 141.4, 135.9, 129.5, 128.7, 128.6, 127.7, 126.5, 123.6, 123.4, 83.2 (*C*(CH_3)$_3$), 60.4 (*C*H-Ph), 60.3 (*C*H_2), 46.3 (*C*H_2), 42.6 (*C*H_2), 28.1 (C(*C*H_3)$_3$) ppm. **LRMS** *m/z* (ES+) m/z: 480 $[M+Na]^+$.

Boc-amidinourea 4g[1]

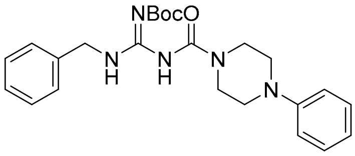

Yield 67% (146 mg). Oil. ^{1}**H NMR** (400 MHz $CDCl_3$) δ 12.31 (br. s., 1H, N*H*), 8.48 (br. s., 1H, N*H*), 7.34-7.25 (m, 7H, *Phenyl*), 6.93-6.86 (m, 3H, *Phenyl*), 4.54 (m, 2H, C*H$_2$*Ph), 3.86 (m, 2H, C*H$_2$*), 3.72 (m, 2H, C*H$_2$*), 3.14 (m, 2H, C*H$_2$*), 3.05 (m, 2H, C*H$_2$*), 1.47 (s, 9H, C(C*H$_3$*)$_3$) ppm. ^{13}**C NMR** (100 MHz $CDCl_3$) δ 162.8 (C=O), 154.5 (C=O), 153.1 (C=N), 151.4, 138.3, 129.2, 128.7, 127.5, 127.4, 120.1, 116.5, 82.5 (*C*(CH_3)$_3$), 49.6 (*2xC*H_2), 49.5 (*2xC*H_2), 44.8 (*C*H_2Ph), 28.1 (C(*C*H_3)$_3$) ppm. **LRMS** *m/z* (ES+) m/z: 460 $[M+Na]^+$.

Boc-amidinourea 4h[1]

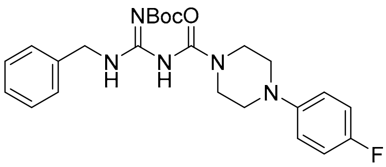

Yield 62% (141 mg). Oil. ^{1}**H NMR** (400 MHz $CDCl_3$) δ 8.46 (br, 1H, N*H*), 7.33-7.25 (m, 5H, *Phenyl*), 6.97-6.93 (m, 2H, *Phenyl*), 6.87-6.84 (m, 2H, *Phenyl*), 4.55 (d, 2H, J = 5.50 Hz, C*H$_2$*Ph), 3.84 (m, 2H, C*H$_2$*-piperazine), 3.70 (m, 2H, C*H$_2$*-piperazine), 3.03 (m, 2H, C*H$_2$*-piperazine), 2.95 (m, 2H, C*H$_2$*-piperazine), 1.45 (s, 9H, C(C*H$_3$*)$_3$) ppm. ^{13}**C NMR** (100 MHz $CDCl_3$) δ 162.8 (C=O), 154.5 (C=O), 153.4 (C=N), 148.1, 138.3, 128.7, 127.5, 127.4, 118.5, 118.4, 115.7, 115.4, 82.6 (*C*(CH_3)$_3$), 50.6 (*2xC*H_2), 44.8 (*C*H_2), 44.7 (*C*H_2), 42.1 (*C*H_2Ph), 28.1 (C(*C*H_3)$_3$) ppm. **LRMS** *m/z* (ES+) m/z: 478 $[M+Na]^+$.

Boc-amidinourea 4i[1]

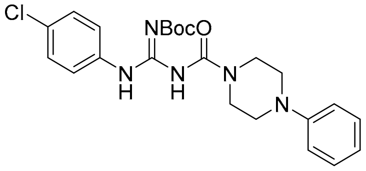

Yield 65% (148 mg). Oil. ^{1}**H NMR** (400 MHz $CDCl_3$) δ 7.52-7.45 (m, 3H, *Phenyl*), 7.29-7.23 (m, 4H, *Phenyl*), 6.92-6.90 (m, 2H, *Phenyl*), 3.85 (m, 2H, C*H$_2$*), 3.73 (m, 2H, C*H$_2$*), 3.14 (m, 2H, C*H$_2$*), 3.10 (m, 2H, C*H$_2$*), 1.49 (s, 9H, C(C*H$_3$*)$_3$) ppm. ^{13}**C NMR** (100 MHz $CDCl_3$) δ 162.6 (C=O), 153.6 (C=O), 152.0 (C=N), 151.2, 135.9, 129.6, 129.2, 128.7, 123.6, 120.2, 116.6, 83.3 (*C*(CH_3)$_3$), 49.7 (*C*H_2), 49.5 (*C*H_2), 44.7 (*C*H_2), 42.3 (*C*H_2), 28.1 (C(*C*H_3)$_3$) ppm. **LRMS** *m/z* (ES+) m/z: 480 $[M+Na]^+$.

Boc-amidinourea 4j[1]

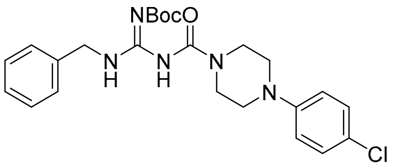

Yield 68% (160 mg). Oil. **^{1}H NMR** (400 MHz $CDCl_3$) δ 8.48 (br, 1H, N*H*), 7.34-7.25 (m, 5H, *Phenyl*), 7.19 (d, 2H, *J* = 8.0 Hz, *Phenyl*), 6.81 (d, 2H, J = 8.0 Hz, *Phenyl*), 4.55 (d, 2H, *J* = 5.5 Hz, CH_2Ph), 3.83 (m, 2H, CH_2-piperazine), 3.69 (m, 2H, CH_2-piperazine), 3.08 (m, 2H, CH_2-piperazine), 2.99 (m, 2H, CH_2-piperazine), 1.45 (s, 9H, C(CH_3)$_3$) ppm. **^{13}C NMR** (100 MHz $CDCl_3$) δ 162.6 (C=O), 153.5 (C=O), 152.5 (C=N), 152.2, 135.6, 129.1, 128.8, 128.3, 123.7, 119.2, 114.1, 110.5, 83.4 (*C*(CH_3)$_3$), 48.6 (*C*H_2), 48.4 (*C*H_2), 44.2 (*C*H_2), 41.8 (*C*H_2), 28.1 (C(*C*H_3)$_3$) ppm. **LRMS** *m/z* (ES+) m/z: 494 $[M+Na]^+$.

Boc-amidinourea 4k[1]

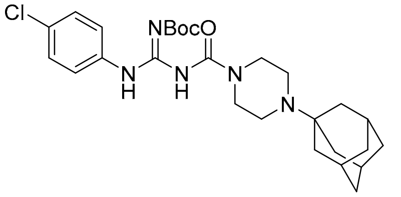

Yield 58% (149 mg). Oil. **^{1}H NMR** (400 MHz $CDCl_3$) δ 7.46 (d, 2H, *J* = 8.0 Hz, *Phenyl*), 7.26 (d, 2H, *J* = 8.0 Hz, *Phenyl*), 3.67 (m, 2H, *C*H_2), 3.55 (m, 2H, *C*H_2), 2.60-2.55 (m, 4H, *2xC*H_2), 2.08 (m, 2H, *Adamantyl*), 1.66-1.55 (m, 13H, *Adamantyl*), 1.47 (s, 9H, C(CH_3)$_3$) ppm. **^{13}C NMR** (100 MHz $CDCl_3$) δ 162.4 (C=O), 153.6 (C=O), 151.7 (C=N), 136.0, 129.3, 128.6, 123.4, 83.1 (*C*(CH_3)$_3$), 54.2 (*C*H_2), 45.5 (*C*H_2), 44.5 (*Adamantyl*), 44.2 (*Adamantyl*), 43.1 (*Adamantyl*), 38.5 (*Adamantyl*), 36.9 (*Adamantyl*), 29.6 (*Adamantyl*), 28.1 (C(*C*H_3)$_3$) ppm. **LRMS** *m/z* (ES+) m/z: 539 $[M+Na]^+$.

General procedure for the synthesis of amidinoureas 5a-k

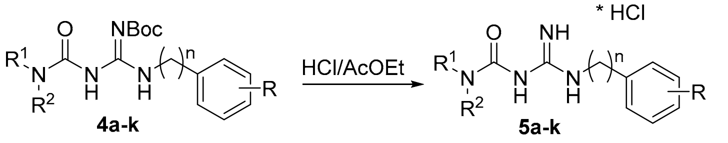

The appropriate amidinourea **4a-k** (0.2 mmol) was placed in a vial. 3 mL of a freshly prepared HCl/AcOEt solution was then added. The mixture was stirred for 48 h. The solvent was then removed under reduced pressure and the product washed several times with cold Et_2O. The desired amidinoureas **5a-k** were obtained as oil HCl salts in quantitative yields.

Amidinourea 5a

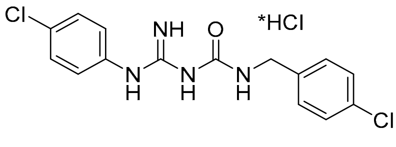

Obtained 73 mg. Oil. **^{1}H NMR** (400 MHz, DMSO-d6): δ 10.87 (br. s., 1H, N*H*), 10.55 (br. s., 1H, N*H*), 8.97 (br. s., 1H, N*H*), 7.50 – 7.56 (m, 2H, *Phenyl*), 7.29 – 7.44 (m, 6H, *Phenyl*), 4.32 (s, 2H, CH_2) ppm. **^{13}C NMR** (101 MHz, DMSO-d6): δ 156.5 (C=N), 154.0 (C=O), 138.1, 133.2, 132.5, 132.1, 130.2,

129.6, 128.8, 128.3, 42.6 (*C*H_2) ppm. **LRMS** *m/z* (ES+) m/z: 337 $[M+H]^+$. **HRMS (ESI):** calcd for $C_{15}H_{15}N_4Cl_2O^+$ ($M + H^+$) 337.0617, found 337.0614.

Amidinourea 5b

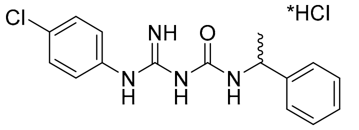

The reaction was carried out on 70 mg (0.17 mmol) of **4b**. Obtained 58 mg. Pale oil. **^{1}H NMR** (400 MHz, CD_3OD): δ 7.52 (m, 2H, *Phenyl*), 7.33 – 7.37 (m, 7H, *Phenyl*), 4.92 (m, 1H, C*H*), 1.50 (d, 3H, C*H_3*) ppm. **^{13}C NMR** (101 MHz, CD_3OD): δ 154.3 (C=N), 152.6 (C=O), 143.0, 134.2, 131.7, 130.0, 128.3, 127.9, 125.6, 49.8 (*C*H), 21.5 (*C*H_3) ppm. **LRMS** *m/z* (ES+) m/z: 317 $[M+H]^+$. **HRMS (ESI):** calcd for $C_{16}H_{18}N_4ClO^+$ ($M + H^+$) 317.1164, found 317.1168.

Amidinourea 5c

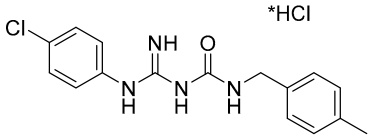

Obtained 69 mg. Oil. **^{1}H NMR** (400 MHz, DMSO-d6): δ 7.51 – 7.56 (m, 2H, *Phenyl*), 7.35 – 7.39 (m, 2H, *Phenyl*), 7.12 – 7.21 (m, 4H, *Phenyl*), 4.27 (s, 2H, C*H_2*), 2.28 (s, 3H, C*H_3*) ppm. **^{13}C NMR** (101 MHz, DMSO-d6): δ 154.1 (C=N), 152.3 (C=O), 136.7, 135.8, 130.2, 129.4, 128.2, 127.8, 43.0 (*C*H_2), 21.1 (*C*H_3) ppm. **LRMS** *m/z* (ES+) m/z: 317 $[M+H]^+$. **HRMS (ESI):** calcd for $C_{16}H_{18}N_4ClO^+$ ($M + H^+$) 317.1164, found 317.1165.

Amidinourea 5d

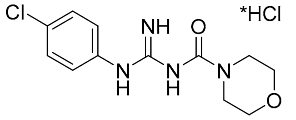

Obtained 62 mg. Oil. **^{1}H NMR** (400 MHz, $CDCl_3$): δ 11.74 (br. s., 1H, N*H*), 9.90 (br. s., 1H, N*H*), 7.47 (m, 2H, *Phenyl*), 7.24 (m, 2H, *Phenyl*), 3.98-3.58 (m, 8H, *Morpholine 4xCH$_2$*) ppm. **^{13}C NMR** (101 MHz, $CDCl_3$): δ 155.8 (C=N), 153.1 (C=O), 130.9, 130.4, 127.4, 66.4 (*2xC*H_2), 31.5 (*2xC*H_2) ppm. **LRMS** *m/z* (ES+) m/z: 283 $[M+H]^+$. **HRMS (ESI):** calcd for $C_{12}H_{16}N_4ClO_2^+$ ($M + H^+$) 283.0956, found 283.0958.

Amidinourea 5e

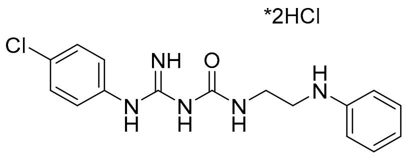

Obtained 79 mg. Dark oil. **^{1}H NMR** (400 MHz, DMSO-d6): δ 10.94 (br. s., 1H, N*H*), 10.51 (br. s., 1H, N*H*), 8.08 (br. s., 1H, N*H*), 7.54 (d, 2H, *J* = 8.5 Hz, *Phenyl-Cl*), 7.29 – 7.39 (m, 5H, *Phenyl*), 7.11 – 7.18 (m, 2H, *Phenyl*), 4.14 (br. s., 1H, N*H*-Ph), 3.42 (m, 2H, (C=O)NHC*H_2*), 3.29 (m, 2H, C*H_2*NHPh) ppm. **^{13}C NMR** (101 MHz, DMSO-d6): δ 154.0 (C=N), 153.3 (C=O), 133.1, 132.6, 130.2, 129.9, 128.2,

120.8, 113.2, 43.6 (CH_2), 38.4 (CH_2) ppm. **LRMS** *m/z* (ES+) m/z: 332 $[M+H]^+$. **HRMS (ESI):** calcd for $C_{16}H_{19}N_5ClO^+$ ($M + H^+$) 332.1273, found 332.1270.

Amidinourea 5f

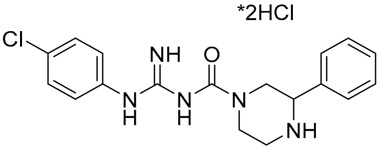

Obtained 84 mg. Oil. ^{1}**H NMR** (400 MHz, DMSO-d6): δ 9.92 (br. s., 1H, N*H*), 7.70 (d, 2H, *J* = 6.5 Hz, *Phenyl-Cl*), 7.44 – 7.56 (m, 5H, *Phenyl*), 7.37 (m, 2H, *Phenyl*), 4.50 (m, 1H, C*H*-piperazine), 3.49 – 3.60 (m, 6H, *3x*C*H*$_2$-piperazine) ppm. ^{13}**C NMR** (101 MHz, DMSO-d6): δ 155.2 (C=N), 154.6 (C=O), 144.5, 137.6, 134.4, 130.1, 129.3, 128.3, 126.6, 123.5, 123.0, 58.4 (*C*H), 58.1 (CH_2), 43.5 (CH_2), 40.1 (CH_2) ppm. **LRMS** *m/z* (ES+) m/z: 358 $[M+H]^+$. **HRMS (ESI):** calcd for $C_{18}H_{21}N_5ClO^+$ ($M + H^+$) 358.1429, found 358.1427.

Amidinourea 5g[1]

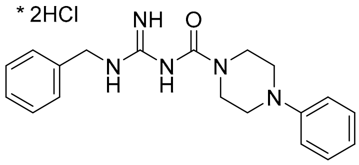

Obtained 80 mg. Yellow oil. ^{1}**H NMR** (400 MHz CDCl$_3$) δ 10.23 45 (br, 1H, N*H*), 9.98 (br, 1H, N*H*), 8.06 (br, 1H, N*H*), 7.30-7.24 (m, 10H, *Phenyls*), 4.53 (m, 2H, C*H*$_2$Ph), 3.52 (m, 4H, *2x*C*H*$_2$ piperazine), 3.31 (m, 4H, *2x*C*H*$_2$ piperazine) ppm. ^{13}**C NMR** (100 MHz CDCl$_3$) δ 155.0 (C=N), 153.0 (C=O), 141.4, 134.5, 130.6, 129.1, 128.4, 127.6, 121.5, 54.8 (*2x*CH$_2$), 45.8 (CH_2), 45.7 (CH_2) ppm. **LRMS** *m/z* (ES+) m/z: 338. **HRMS (ESI):** calcd for $C_{19}H_{24}N_5O^+$ ($M + H^+$) 338.1978, found 338.1974.

Amidinourea 5h[1]

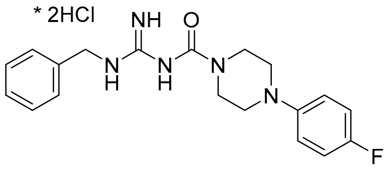

Obtained 83 mg. Yellow oil. ^{1}**H NMR** (400 MHz CDCl$_3$) δ 7.93 (br, 1H, N*H*), 7.34 (m, 6H, *Phenyl*), 7.15 (m, 3H, *Phenyl*), 4.61 (m, 2H, C*H*$_2$Ph), 4.35 (m, 4H, *2x*C*H*$_2$ piperazine), 3.60 (m, 4H, *2x*C*H*$_2$ piperazine) ppm. ^{13}**C NMR** (100 MHz CDCl$_3$) δ 155.6 (C=N), 152.9 (C=O), 140.9, 133.9, 129.4, 128.2, 127.5, 123.9, 117.8, 117.6, 55.2 (CH_2), 53.5 (CH_2), 45.9 (*2x*CH$_2$) ppm. **LRMS** *m/z* (ES+) m/z: 356 $[M+H]^+$. **HRMS (ESI):** calcd for $C_{19}H_{23}FN_5O^+$ ($M + H^+$) 356.1881, found 356.1883.

Amidinourea 5i[1]

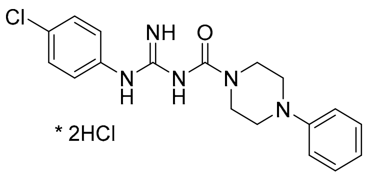

Obtained 85 mg. Yellow oil. ^{1}**H NMR** (400 MHz CD$_3$OD) δ 7.77-7.75 (m, 2H, *Phenyl-Cl*), 7.56-7.48 (m, 5H, *Phenyl*), 7.36-7.34 (m, 2H, *Phenyl-Cl*), 3.78-3.68 (m, 4H, *2x*C*H*$_2$ piperazine), 3.25 (m, 4H, *2x*C*H*$_2$ piperazine) ppm. ^{13}**C NMR** (100 MHz CD$_3$OD) δ 155.6 (C=N), 153.2 (C=O), 142.6, 134.8,

132.5, 131.0, 130.7, 130.5, 128.1, 121.7, 121.5, 54.9 (*2xC*H_2), 49.1 (*2xC*H_2) ppm. **LRMS** *m/z* (ES+) m/z: 358 $[M+H]^+$. **HRMS (ESI):** calcd for $C_{18}H_{21}N_5ClO^+$ ($M + H^+$) 358.1429, found 358.1431.

Amidinourea 5j[1]

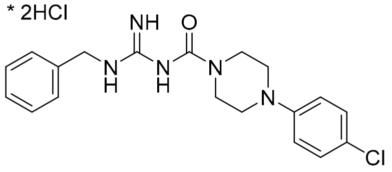

Obtained 85 mg. Yellow oil. **^{1}H NMR** (400 MHz $CDCl_3$) δ 7.66 (br, 1H, N*H*), 7.34-7.25 (m, 9H, *Phenyls*), 4.57 (m, 2H, C*H$_2$*Ph), 3.61-3.38 (m, 8H, *4xCH$_2$* piperazine) ppm. **^{13}C NMR** (100 MHz $CDCl_3$) δ 155.2 (C=N), 153.0 (C=O), 143.0, 134.3, 130.5, 129.2, 128.5, 127.5, 122.4, 53.9 (*C*H_2), 50.6 (*C*H_2), 45.8 (*2xC*H_2) ppm. **LRMS** *m/z* (ES+) m/z: 372 $[M+H]^+$. **HRMS (ESI):** calcd for $C_{19}H_{23}N_5ClO^+$ ($M + H^+$) 372.1586, found 372.1588.

Amidinourea 5k[1]

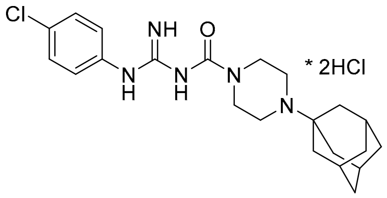

Obtained 95 mg. Yellow oil. **^{1}H NMR** (400 MHz DMSO-d6) δ 7.52-7.50 (m, 2H, *2xCH* Phenyl), 7.38-7.32 (m, 2H, *2xCH* Phenyl), 3.70-3.46 (m, 8H, *4xCH$_2$* piperazine), 2.12 (m, 3H, *Adamantyl*), 1.94 (m, 6H, *Adamantyl*), 1.59 (m, 6H, *Adamantyl*) ppm. **^{13}C NMR** (100 MHz DMSO-d6) δ 155.0 (C=N), 151.9 (C=O), 136.4, 130.3, 129.0, 128.8, 124.8, 64.2 (*2xC*H_2 piperazine), 63.9 (*2xC*H_2 piperazine), 44.1 (*C*-N-adamantyl), 35.8 (*3xC*H_2-adamantyl), 35.6 (*3xC*H_2-adamantyl), 29.4 (*3xC*-adamantyl)ppm. **LRMS** *m/z* (ES+) m/z: 416 $[M+H]^+$. **HRMS (ESI):** calcd for $C_{22}H_{31}N_5ClO^+$ ($M + H^+$) 416.2212, found 416.2210.

Purity check

All the compounds were analysed by LC-MS prior submission for the biological assays and proved to be pure at >95%. The LC-MS analysis was carried out using the following conditions: flow 1.3 mL/min; eluent H_2O/CH_3CN 70:30; column, Thermo Scientific Acclaim 120 C18 Columns, 100mm L x 4.6mm ID, 5.0um.

Compound	Retention time	Compound	Retention time
**5a**	7.29 min[a]	**5g**	15.7 min
**5b**	20.5 min	**5h**	18.3 min
**5c**	24.2 min	**5i**	8.4 min[a]
**5d**	3.8 min	**5j**	9.9 min[a]
**5e**	15.3 min	**5k**	21.6 min
**5f**	6.1 min		

[a]Eluent used is H_2O/CH_3CN 60:40

References

1. A. Magri, R. Reilly, N. Scalacci, M. Radi, M. Hunter, M. Ripoll, A. H. Patel, D. Castagnolo, *Bioorg. Med. Chem. Lett.* **2015**, *25,* 5372-5376.
